# Supplementary material for: High-fidelity spatial mode transmission through a 1-km-long multimode fiber via vectorial time reversal
Source: Nat Commun. 2021 Mar 25;12:1866. doi: 10.1038/s41467-021-22071-w (PMC7994418; doi:10.1038/s41467-021-22071-w)
Supplement: Supplementary file 1 — Supplementary Information [file 41467_2021_22071_MOESM1_ESM.pdf]

High-fidelity spatial mode transmission through a 1-km-long multimode fiber via vectorial time reversal: supplementary information

Yiyu Zhou[1,*], Boris Braverman[2], Alexander Fyffe[3], Runzhou Zhang[4], Jiapeng Zhao[1], Alan E. Willner[4], Zhimin Shi[3] and Robert W. Boyd[1,2]

[1]The Institute of Optics, University of Rochester, Rochester, New York 14627, USA
[2]Department of Physics, University of Ottawa, Ottawa, Ontario K1N 6N5, Canada
[3]Department of Physics, University of South Florida, Tampa, FL 33620, USA
[4]Department of Electrical Engineering, University of Southern California, Los Angeles, California, 90089, USA
[*]Corresponding author: yzhou62@ur.rochester.edu

Supplementary Note 1 — Literature review

No.	Method	Multimode fiber length	Number of demonstrated modes	Reference
1	Vectorial time reversal	1 km	210 HG/LG modes	This work
2	Transfer matrix inversion	2 m	6 discrete spot modes	[1]
3	Transfer matrix inversion	5 m	N/A	[2]
4	Transfer matrix inversion	2 m	110 LP modes	[3]
5	Transfer matrix inversion	0.3 m	110 LP modes	[4]
6	Transfer matrix inversion	0.5 m	18 discrete spot modes	[5]
7	Transfer matrix inversion	2 m	N/A	[6]
8	Transfer matrix inversion	1 m	N/A	[7]
9	Transfer matrix inversion	2 m	N/A	[8]
10	Transfer matrix inversion	1 m	N/A	[9]
11	Scalar time reversal	1 m	N/A	[10]
12	Scalar time reversal	0.19 m	N/A	[11]
13	Scalar time reversal	0.3 m	N/A	[12]
14	Scalar time reversal	2 m	N/A	[13]
15	Mode-group excitation	44.3 km	6 mode groups	[14]
16	Mode-group excitation	5 km	8 mode groups	[15]
17	Mode-group excitation	2.6 km	4 mode groups	[16]

Supplementary Table 1. Summary of methods for modal crosstalk suppression in standard multimode fibers. It can be seen that transfer matrix inversion has only been applied to short fibers that are less than 5-m long. This is because the characterization of a high-dimensional transfer matrix can take as long as hours. Since it is technically challenging to stabilize long fibers for hours, short fibers are used for experimental demonstrations. It can also be seen that scalar time reversal has only been used for short fibers. In Supplementary Note 6 we show that scalar time reversal cannot be used for a 1-km-long fiber due to the inevitable polarization mixing in long fibers. Mode-group excitation can be used to transmit mode groups over a long fiber. However, a standard MMF only supports a few mode groups, and thus this method is not comparable to our approach. HG: Hermite-Gauss. LG: Laguerre-Gauss. LP: linearly polarized.

Supplementary Note 2 — Experimental setup

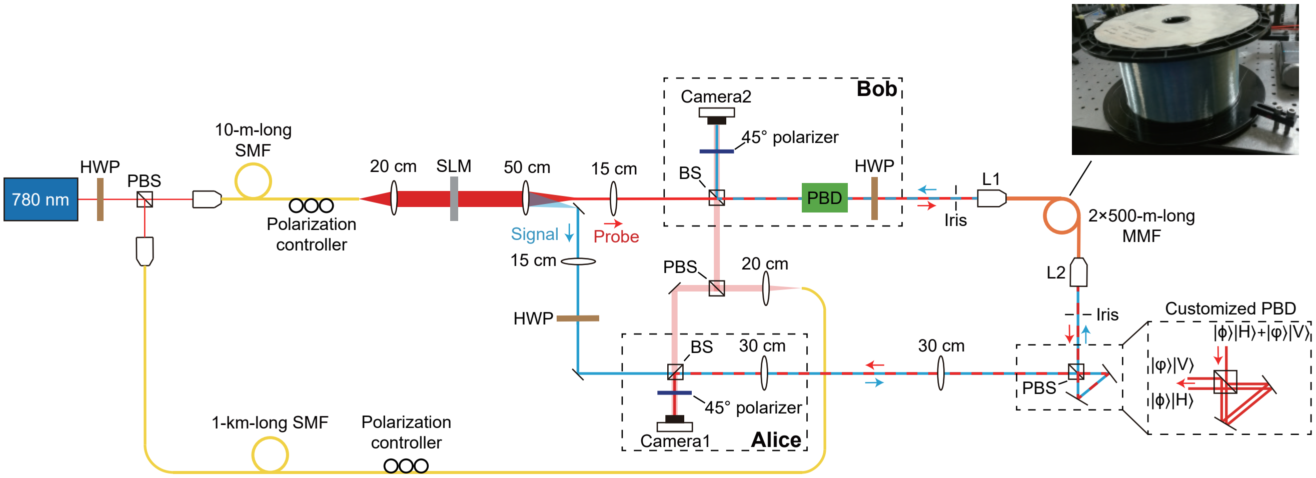

Supplementary Figure 1. The schematic of the experimental setup. HWP: half-wave plate. PBS: polarizing beamsplitter. SMF: single-mode fiber. SLM: spatial light modulator. BS: beamsplitter. PBD: polarizing beam displacer. MMF: multimode fiber. The same SLM is used to generate probe beams for Bob (denoted by red lines) and time-reversed signal beams (denoted by blue lines) for Alice by switching the phase grating. The multimode fiber spool is shown at the upper right corner and is resting on the optical table without any specialized thermal or mechanical stabilization. The optical table used in the experiment is not floated. The customized PBD is made of a PBS and two mirrors as illustrated by the inset.

The detailed experimental setup is shown in Supplementary Fig. 1. A 780 nm laser (DL pro, Toptica) is used as the light source. The light is spatially filtered by a 10-m-long single-mode fiber (SMF) and then collimated to illuminate the spatial light modulator (SLM). A single SLM (Pluto 2 VIS-020, Holoeye) is used to generate both the probe beam for Bob and the signal beam for Alice with the choice being made by switching the overall phase grating written onto the SLM. A binary phase grating is used to generate complex-amplitude spatial modes in the first diffraction order [17]. The probe beam generated by Bob is denoted by red lines and the signal beams generated by Alice is denoted by blue lines in Supplementary Fig. 1. Bob uses a polarizing beam displacer (MBDA10, Karl Lambrecht) to generate a horizontally polarized spatial mode, and the polarization can be adjusted by a subsequent half-wave plate (HWP). The generated probe beam is then coupled into a 1-km-long multimode fiber (MMF) by an aspheric lens L1 (C110TMD-B, Thorlabs). The spatial mode beam waist size in the MMF used in our experiment is $w_0 = 5.06$ μm [18]. The MMF is comprised of two 500-m-long fibers (Clearcurve OM3, Corning) that are spliced together. The scrambled probe beam received by Alice is collimated by an aspheric lens L2 (C230TMD-B, Thorlabs), and a subsequent Sagnac interferometer is used as a customized polarizing beam displacer to coherently separate the horizontal and vertical polarization components of the scrambled probe beam to two beams that propagate along the same direction but are displaced with respect to each other [19]. The Sagnac interferometer as a customized polarizing beam displacer (PBD) provides more flexibility than the commercially available polarizing beam displacer because the transverse separation between the two displaced beams can be tuned by adjusting the mirrors in the Sagnac interferometer. A 1-km-long SMF is used to provide a coherent reference light source to interfere with the scrambled probe beam. A digital camera (Camera 1, BFS-U3-16S2M-CS,

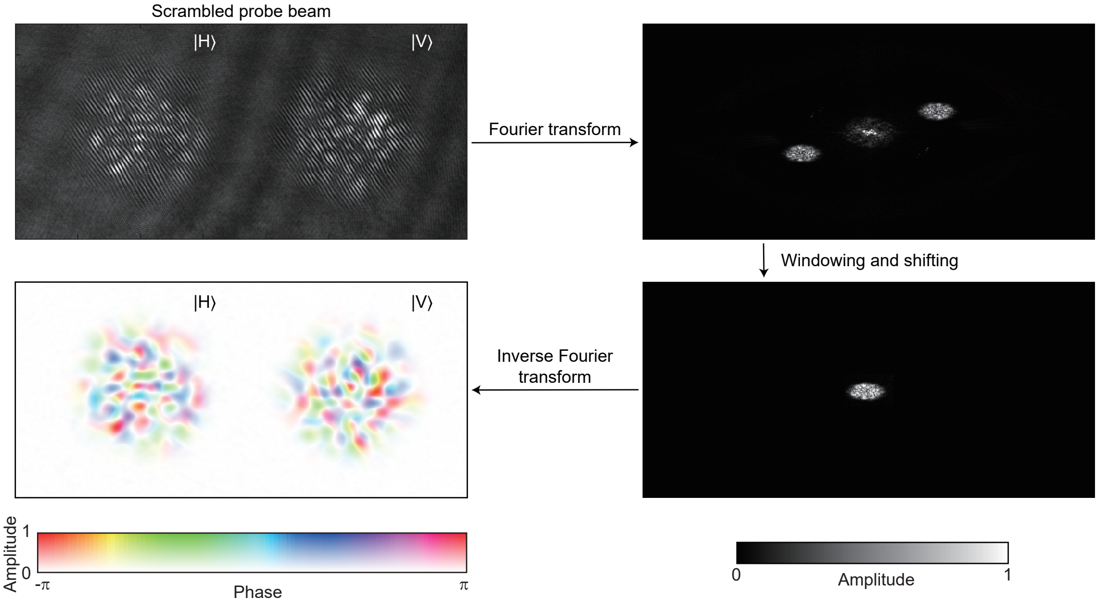

Supplementary Figure 2. Procedure for vectorial off-axis holography. The horizontally polarized beam and the vertically polarized beam are displaced by a PBD and then interfere with a 45° polarized beam (see Fig. 1 in the manuscript). The interference pattern is recorded by a camera. A Fourier transform is performed and the first-order component is selected and shifted to the image center. Then an inverse Fourier transform is performed to retrieve the polarization, amplitude, and phase of the scrambled probe beam.

FLIR) is used by Alice to measure the interference pattern and perform the off-axis holography [20]. In this way, Alice can measure the amplitude, phase, and polarization of the scrambled probe beam via a single-shot measurement. The time-reversed signal beam is generated by the SLM and then directed back to the MMF. More details about the detection and generation of vector beam can be found in [21], and the alignment procedure for time reversal can be found in [22]. Bob measures the unscrambled signal beam by another camera (Camera 2, BFS-U3-31S4M-C, FLIR) and performs the digital spatial mode decomposition to obtain the crosstalk matrix.

In our experiment, both Alice and Bob perform data processing for off-axis holography in MATLAB on a desktop computer (Intel i7-9700K with Nvidia RTX 2070 Super). The digital signal processing for off-axis holography involves several fast Fourier transforms (FFTs), which can be significantly sped up by using a dedicated digital signal processor. The procedure of vectorial off-axis holography is shown in Supplementary Fig. 2. The horizontally polarized component and the vertically polarized component interfere with a 45° polarized plane wave, and the interference pattern is recorded by a camera. Then a Fourier transform is performed, and the first-order component in the Fourier domain is selected and shifted to the center. Finally an inverse Fourier transform is performed, and thus the amplitude and phase of the scrambled probe beam are obtained. Ideally, the digital signal processing for Alice can even be avoided if the camera for off-axis holography and the SLM for generating the time-reversed signal beam are exactly placed at positions that are imaging planes with respect to each other [22]. When the positions of camera and SLM are perfectly aligned, we can directly imprint the digital interference pattern recorded by the camera onto the SLM without performing any digital signal processing. Instead of carefully aligning the position of the camera,

Alice digitally compensates the misalignments such as tip and tilt, transverse and axial displacement, and defocus (see [22]). At Bob's side, we are using a second digital off-axis holography to measure the unscrambled signal beam, which allows us to obtain the crosstalk matrix with reduced experimental complexity. It should be noted that the off-axis holography can be readily replaced by a spatial mode sorter [23, 24, 25] to analyze the crosstalk matrix and enable high-speed spatial mode detection.

Supplementary Note 3 — Definition of the single mode index

The Laguerre-Gauss and Hermite-Gauss mode are typically denoted by two indices, which are (p,ℓ) for Laguerre-Gauss mode and (m,n) for Hermite-Gauss mode. For the convenience of presentation we use a single mode index $j = 0, 1, 2, \cdots$ to denote the modes. Here we refer to the convention of Zernike polynomials [26] and adopt the following definitions. For a specific mode index j, the mode group number N can be calculated as $N = \text{ceil}((-3+\sqrt{9+8j})/2)$. Then we have $\ell = 2j - N(N+2)$ and $p = (N-|\ell|)/2$ for Laguerre-Gauss mode. Given the one-to-one correspondence between Hermite-Gauss mode and Laguerre-Gauss mode [27], we can define $m = p + \max(\ell, 0)$ and $n = p - \min(\ell, 0)$ for Hermite-Gauss mode. On the other hand, for a given (p,ℓ) or (m,n), we can calculate j as $j = [N(N+2)+\ell]/2$ with $N = 2p + |\ell|$ for Laguerre-Gauss modes and $j = [N(N+2)+m-n]/2$ with $N = m+n$ for Hermite-Gauss modes. Examples of the conversion relation between j, N, (p,ℓ) and (m,n) are given in Supplementary Table. 2.

j	0	1	2	3	4	5
N	0	1	1	2	2	2
p	0	0	0	0	1	0
ℓ	0	-1	1	-2	0	2
m	0	0	1	0	1	2
n	0	1	0	2	1	0

Supplementary Table 2. Examples of relation between the single mode index j, mode group number N, Laguerre-Gauss mode indices (p,ℓ), and Hermite-Gauss mode indices (m,n).

Supplementary Note 4 — Crosstalk matrix and the normalized modal fidelity

The 210×210 unnormalized crosstalk matrix of the scrambled probe beams received by Alice when Bob transmits standard Laguerre-Gauss and Hermite-Gauss modes (i.e. in the absence of vectorial time reversal) are shown in Supplementary Fig. 3 and Supplementary Fig. 4. Due to the strong spatial mode scrambling, the average unnormalized modal fidelity in this case is ≈1% for both Laguerre-Gauss modes and Hermite-Gaussian modes. The 210×210 unnormalized crosstalk matrix in the presence of vectorial time reversal are presented in Supplementary Fig. 5 and Supplementary Fig. 6. The crosstalk matrix is calculated as follows. The received vectorial mode is denoted as $|\phi\rangle = |\psi_1, \mathrm{H}\rangle + |\psi_2, \mathrm{V}\rangle$, where H and V represent the horizontal and vertical polarization state, ψ_1 and ψ_2 represent the corresponding spatial mode, and $|\phi\rangle$ is normalized such that $\langle\phi|\phi\rangle = 1$. Each element in the crosstalk matrix is the squared inner product between the received mode $|\phi\rangle$ and a particular Laguerre-Gauss or Hermite-Gauss mode. As an example, for a horizontally polarized Laguerre-Gauss mode $|\mathrm{LG}_j, \mathrm{H}\rangle$, the squared inner product can be expressed as $|\langle\phi|\mathrm{LG}_j, \mathrm{H}\rangle|^2$, where $0 \leq j \leq 104$ is the single mode index. For a Laguerre-Gauss mode, it is normalized such that $\langle\mathrm{LG}_j, \mathrm{H}|\mathrm{LG}_j, \mathrm{H}\rangle = 1$ and $\langle\mathrm{LG}_j, \mathrm{V}|\mathrm{LG}_j, \mathrm{V}\rangle = 1$. Similar normalization is also applied to Hermite-Gauss modes. It should be noted that since the spatial modes with $0 \leq j \leq 104$ do not form a complete basis set, the sum of each row in the crosstalk matrix is less than unity, *i.e.* $\sum_{j=0}^{104} |\langle\phi|\mathrm{LG}_j, \mathrm{H}\rangle|^2 + |\langle\phi|\mathrm{LG}_j, \mathrm{V}\rangle|^2 < 1$. However, we didn't normalize the crosstalk matrix and directly present the unnormalized modal fidelity in the manuscript. After normalizing the sum of each row of the crosstalk matrix to unity, the modal fidelity can be higher as shown in Supplementary Fig. 7. It can be seen that the average of normalized modal fidelity is 91.5% for Laguerre-Gauss modes and 89.3% for Hermite-Gauss modes. This is because the crosstalk due to coupling to higher-order modes ($j \geq 105$) is discarded. This is permissible in an experiment because the higher-order modes can in principle be separated by a mode sorter in practical applications and thus does not contribute to the crosstalk.

To aid readers for analyzing the crosstalk matrix, we also present the crosstalk distributions in the following four categories. Here we assume the mode of interest is a horizontally polarized Laguerre-Gauss mode $|\mathrm{LG}_j, \mathrm{H}\rangle$ and the received time-reversed mode is $|\phi\rangle = |\psi_1, \mathrm{H}\rangle + |\psi_2, \mathrm{V}\rangle$ as an example. The four crosstalk categories are (1) crosstalk from coupling to modes inside the crosstalk matrix with degenerate polarization, which can be expressed as $C_1 = \sum_k |\langle\phi|\mathrm{LG}_k, \mathrm{H}\rangle|^2$ for $0 \leq k \leq 104$ and $k \neq j$. (2) crosstalk from coupling to modes inside the crosstalk matrix with orthogonal polarization, which can be expressed as $C_2 = \sum_k |\langle\phi|\mathrm{LG}_k, \mathrm{V}\rangle|^2$ for $0 \leq k \leq 104$. (3) crosstalk from coupling to modes outside the crosstalk matrix with degenerate polarization, which can be expressed as $C_3 = \sum_k |\langle\phi|\mathrm{LG}_k, \mathrm{H}\rangle|^2$ for $k \geq 105$ or equivalently $C_3 = |\langle\psi_1|\psi_1\rangle|^2 - C_1 - |\langle\psi_1|\mathrm{LG}_j\rangle|^2$. (4) crosstalk from coupling to modes outside the crosstalk matrix with orthogonal polarization, which can be expressed as $C_4 = \sum_k |\langle\phi|\mathrm{LG}_k, \mathrm{V}\rangle|^2$ for $k \geq 105$ or equivalently $C_4 = |\langle\psi_2|\psi_2\rangle|^2 - C_2$. These results are shown in Supplementary Fig. 8.

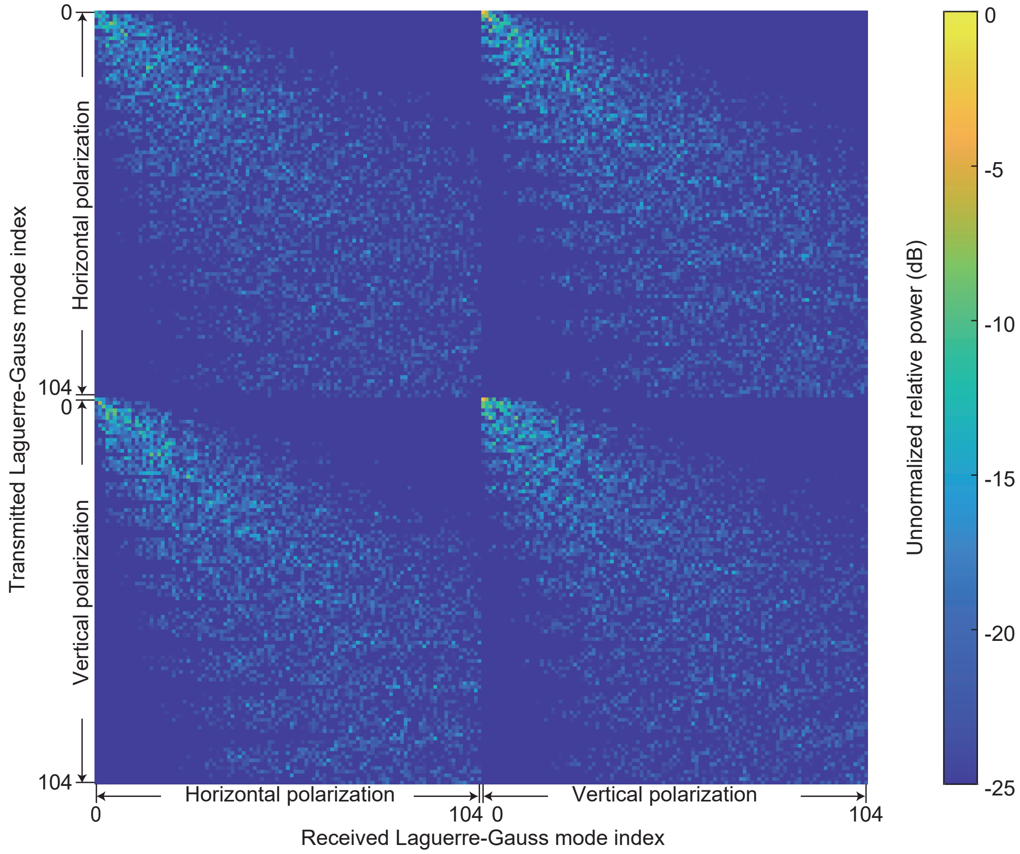

Supplementary Figure 3. Unnormalized 210×210 crosstalk matrix on a logarithmic scale for scrambled probe beams received by Alice when Bob transmits Laguerre-Gauss modes in the absence of vectorial time reversal.

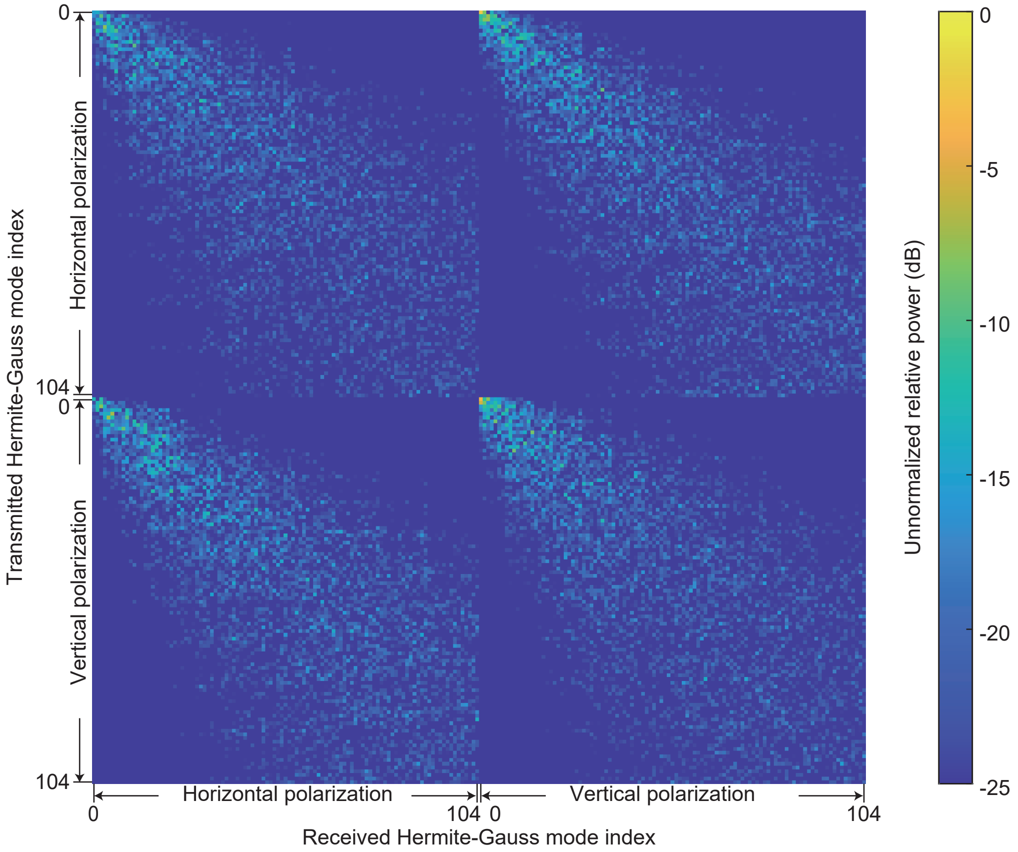

Supplementary Figure 4. Unnormalized 210×210 crosstalk matrix on a logarithmic scale for scrambled probe beams received by Alice when Bob transmits Hermite-Gauss modes in the absence of vectorial time reversal.

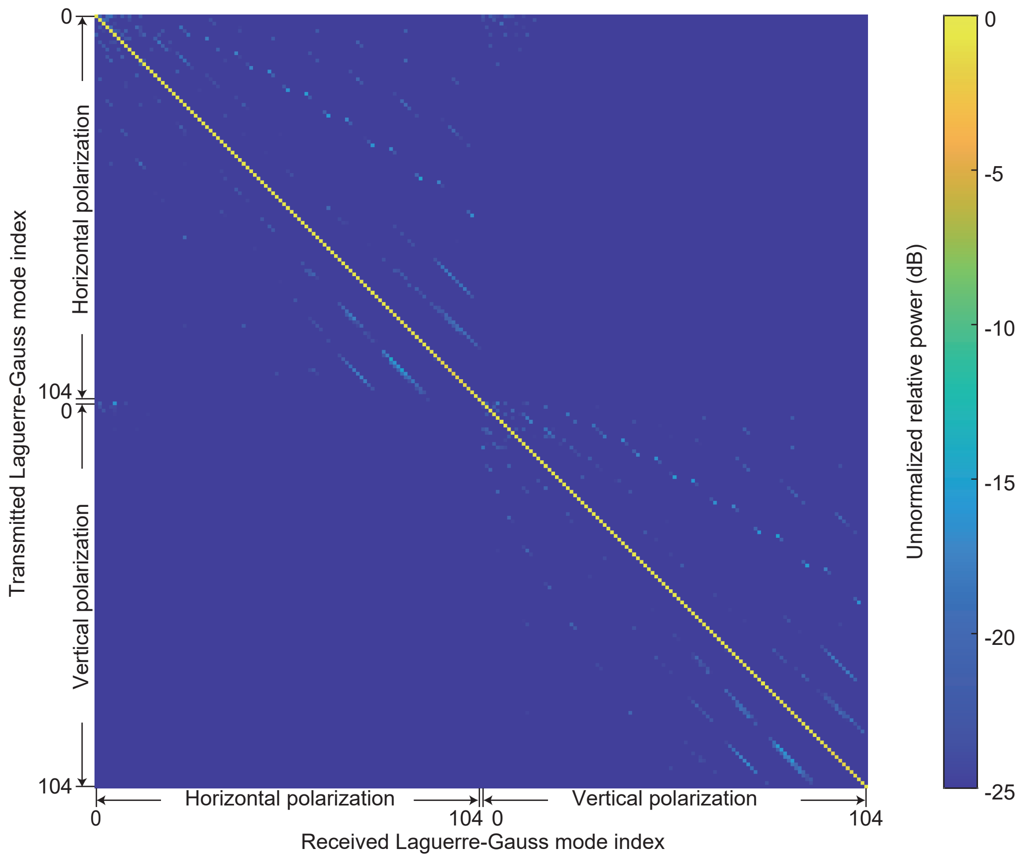

Supplementary Figure 5. Unnormalized 210×210 crosstalk matrix on a logarithmic scale for unscrambled Laguerre-Gauss modes received by Bob when performing vectorial time reversal.

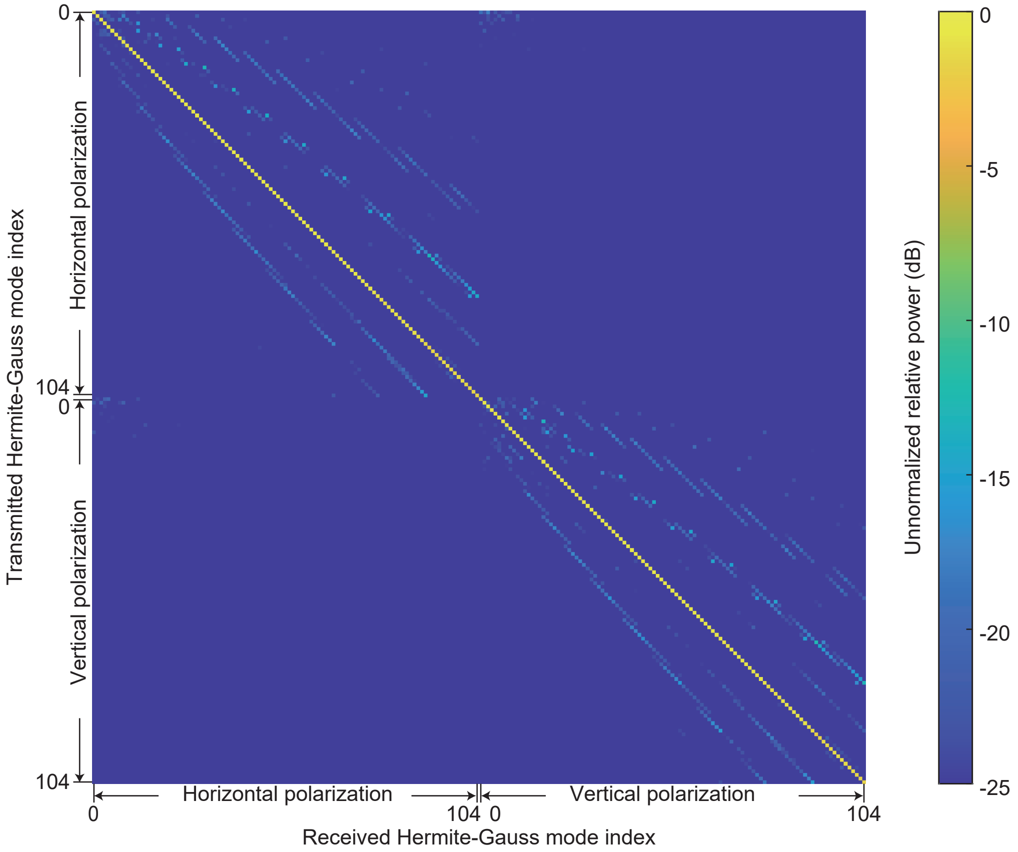

Supplementary Figure 6. Unnormalized 210×210 crosstalk matrix on a logarithmic scale for unscrambled Hermite-Gauss modes received by Bob when performing vectorial time reversal.

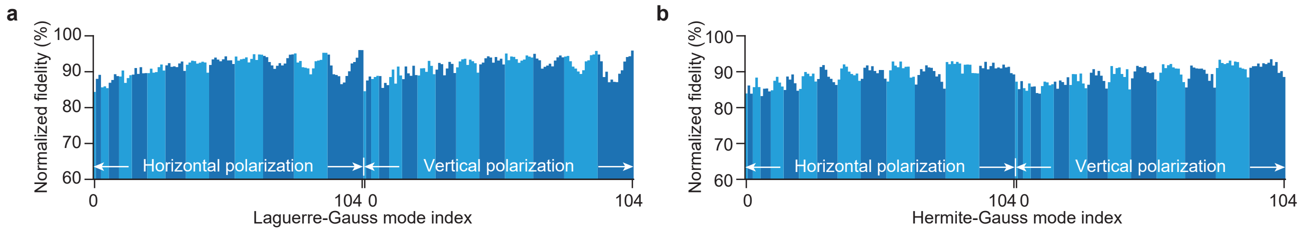

Supplementary Figure 7. Normalized modal fidelity for unscrambled (**a**) Laguerre-Gauss and (**b**) Hermite-Gauss modes. The normalized modal fidelity is calculated by dividing the corresponding diagonal element (i.e. the unnormalized modal fidelity) of the crosstalk matrices (shown in Supplementary Fig. 5 for Laguerre-Gauss modes and Supplementary Fig. 6 for Hermite-Gauss modes) by the sum of elements in each column.

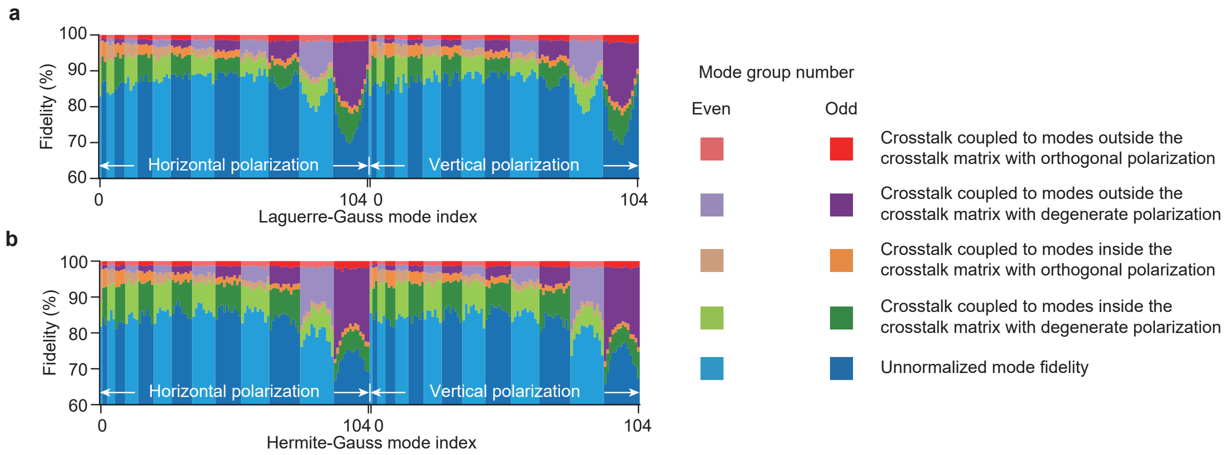

Supplementary Figure 8. The modal fidelity and crosstalk distributions for unscrambled (**a**) Laguerre-Gauss modes and (**b**) Hermite-Gauss modes.

Supplementary Note 5 — Experimental generation fidelity of SLM

In the manuscript we show that the average modal fidelity without normalization is 85.6% for Laguerre-Gauss modes and 82.6% for Hermite-Gauss modes. The main reason for imperfect modal fidelity is attributed to the imperfect spatial mode generation fidelity of the SLM. In the experiment, Alice uses a SLM to generate the phase conjugate of the scrambled probe beam, and Bob uses a SLM to generate the Laguerre-Gauss or Hermite-Gauss modes. We characterize the fidelity of the generated spatial modes and horizontal polarization component of scrambled probe beams using off-axis holography, with the results shown in Supplementary Fig. 9. We also measure the complex field of the generated signal beam $|\psi_s\rangle$, and calculate the overlap integral with the scrambled probe beam $|\psi_p\rangle$ to get the signal beam fidelity as $|\langle\psi_s|\psi_p\rangle|^2$. We take the product of the probe spatial modal fidelity and the signal beam fidelity as the experimental generation fidelity, which is a simple estimate of time-reversed modal fidelity. In Supplementary Fig. 9(a-d) we show the ideal and generated spatial mode and time-reversed signal beam for HG(2,5) and LG(1,4) modes. In Supplementary Fig. 2(c,d) in the manuscript we present the experimental generation fidelity for individual Laguerre-Gauss and Hermite-Gauss modes, which shows reasonable agreement with the fidelity of time-reversed modes. In general, the modal fidelity for Laguerre-Gauss modes is slightly higher than that of Hermite-Gauss modes, and the Laguerre-Gauss modes with $\ell \neq 0$ mode have a higher fidelity than those with $\ell = 0$. In addition, the signal beam generation has a slightly lower fidelity than the well-defined Laguerre-Gauss and Hermite-Gauss modes. The maximum experimental generation fidelity we measured is 90.8% for Laguerre-Gauss modes and 88.4% for Hermite-Gauss modes, and the average experimental generation fidelity is 85.8% for Laguerre-Gauss modes and 83.7% for Hermite-Gauss modes. Thus, by improving the experimental generation fidelity through using a higher-quality SLM, the performance of vectorial time reversal can be further enhanced.

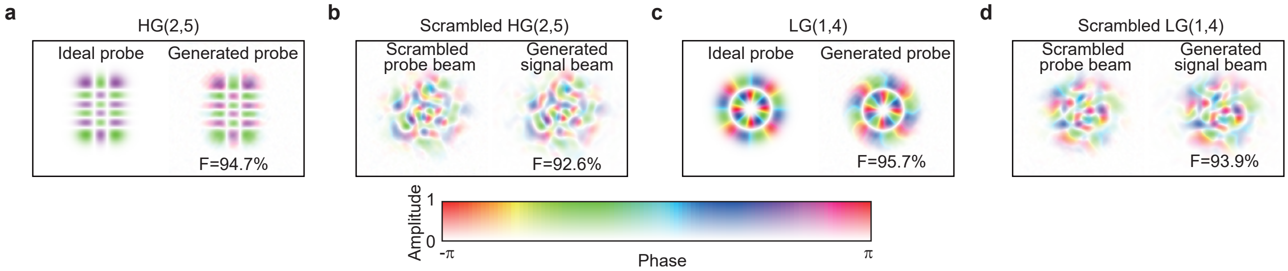

Supplementary Figure 9. (**a**–**d**) The mode generation fidelity for HG(2,5) and LG(1,4) modes. The HG(2,5) and LG(1,4) probe beams generated by SLM are shown at the right panel of (**a**) and (**c**), and the corresponding ideal modes are shown at the left panel for comparison. The corresponding phase conjugate of the scrambled probe beams are shown at the left panel of (**b**) and (**d**), and the generated signal beams are shown at the right panel. The calculated fidelity (F) is listed below the generated modes.

Supplementary Note 6 — Experimental results of scalar time reversal

Here we show the results for scalar time reversal. Bob transmits horizontally polarized spatial modes to Alice. While Alice can measure both the horizontal and vertical polarization components of the scrambled probe beam, she only generates the phase conjugate of the horizontal polarization and ignores the vertical polarization. The experimental results are shown in Supplementary Fig. 10. It can be seen that the modal fidelity of scalar time reversal is significantly worse than that of vectorial time reversal. The average modal fidelity is 41.2% for Laguerre-Gaussian modes and 39.7% for Hermite-Gauss modes. The results here confirm the necessity of vectorial time reversal.

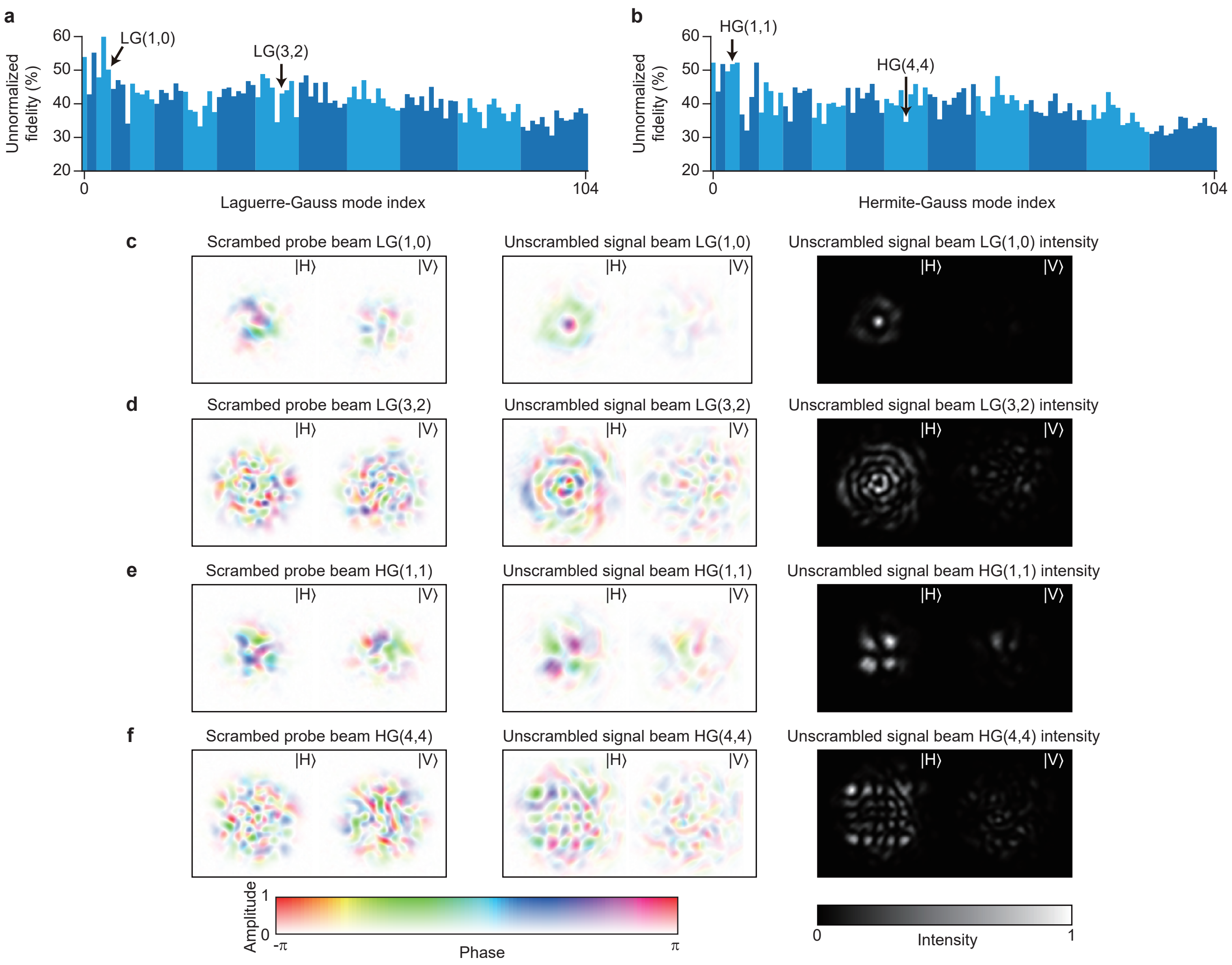

Supplementary Figure 10. (**a**, **b**) The measured modal fidelity for unscrambled Laguerre-Gauss modes and Hermite-Gauss modes received by Bob when Alice performs scalar time reversal (i.e. using one polarization only). (**c**–**f**) The measured scrambled probe beam, the unscrambled signal beam, and the corresponding intensity for LG(1,0), LG(3,2), HG(1,1), HG(4,4) modes respectively.

Supplementary Note 7 — Polarization crosstalk matrix

In the manuscript we show the normalized polarization crosstalk matrix within the individual spatial mode subspace (see Fig. 3a in the manuscript). Here we continue to use LG_j to show how the calculation is performed. For the received time-reversed mode $|\phi\rangle$, we first calculate the unnormalized squared inner product for horizontally (H), vertically (V), diagonally (D), and anti-diagonally (A) polarized LG_j mode as $F_H^u = |\langle\phi|\mathrm{LG}_j,\mathrm{H}\rangle|^2$, $F_V^u = |\langle\phi|\mathrm{LG}_j,\mathrm{V}\rangle|^2$, $F_D^u = |\langle\phi|\mathrm{LG}_j,\mathrm{D}\rangle|^2$, and $F_A^u = |\langle\phi|\mathrm{LG}_j,\mathrm{A}\rangle|^2$, where $|\mathrm{D}\rangle = (|\mathrm{H}\rangle + |\mathrm{V}\rangle)/\sqrt{2}$ and $|\mathrm{A}\rangle = (|\mathrm{H}\rangle - |\mathrm{V}\rangle)/\sqrt{2}$. Then the normalized fidelity can be calculated as $F_\mathrm{H} = F_\mathrm{H}^\mathrm{u}/(F_\mathrm{H}^\mathrm{u} + F_\mathrm{V}^\mathrm{u})$, $F_\mathrm{V} = F_\mathrm{V}^\mathrm{u}/(F_\mathrm{H}^\mathrm{u} + F_\mathrm{V}^\mathrm{u})$, $F_\mathrm{D} = F_\mathrm{D}^\mathrm{u}/(F_\mathrm{D}^\mathrm{u} + F_\mathrm{A}^\mathrm{u})$, and $F_\mathrm{A} = F_\mathrm{A}^\mathrm{u}/(F_\mathrm{D}^\mathrm{u} + F_\mathrm{A}^\mathrm{u})$. These four numbers form one row of the crosstalk matrix, and the rest of the matrix can be similarly calculated. The normalized polarization crosstalk matrix for Hermite-Gauss modes is also calculated in this way. Since the calculation is performed for a (4×4)-dimensional state space, the crosstalk is small. It should be noted that such calculation is permissible because in an experiment one can use a polarization-independent spatial mode sorter and a polarizing beamsplitter to access the (4×4)-dimensional state space, and polarization crosstalk that couples to other spatial modes can be discarded.

Supplementary References

1. Valencia, N. H., Goel, S., McCutcheon, W., Defienne, H. & Malik, M. Unscrambling entanglement through a complex medium. *Nat. Phys.* **16**, 1112–1116 (2020).

2. Mounaix, M. et al. Time reversed optical waves by arbitrary vector spatiotemporal field generation. *Nat. Commun.* **11**, 5813 (2020).

3. Carpenter, J., Eggleton, B. J. & Schröder, J. 110x110 optical mode transfer matrix inversion. *Opt. Express* **22**, 96–101 (2014).

4. Plöschner, M., Tyc, T. & Čižmár, T. Seeing through chaos in multimode fibres. *Nat. Photon.* **9**, 529 (2015).

5. Leedumrongwatthanakun, S. et al. Programmable linear quantum networks with a multimode fibre. *Nat. Photon.* **14**, 139–142 (2020).

6. Gordon, G. S. et al. Characterizing optical fiber transmission matrices using metasurface reflector stacks for lensless imaging without distal access. *Phys. Rev. X* **9**, 041050 (2019).

7. Mounaix, M. & Carpenter, J. Control of the temporal and polarization response of a multimode fiber. *Nat. Commun.* **10**, 5085 (2019).

8. Xiong, W. et al. Complete polarization control in multimode fibers with polarization and mode coupling. *Light Sci. Appl.* **7**, 54 (2018).

9. Xiong, W., Hsu, C. W. & Cao, H. Long-range spatio-temporal correlations in multimode fibers for pulse delivery. *Nat. Commun.* **10**, 2973 (2019).

10. Papadopoulos, I. N., Farahi, S., Moser, C. & Psaltis, D. Focusing and scanning light through a multimode optical fiber using digital phase conjugation. *Opt. Express* **20**, 10583–10590 (2012).

11. Ma, C. et al. Structured light beams created through a multimode fiber via virtual Fourier filtering based on digital optical phase conjugation. *Appl. Opt.* **59**, 701–705 (2020).

12. Morales-Delgado, E. E., Farahi, S., Papadopoulos, I. N., Psaltis, D. & Moser, C. Delivery of focused short pulses through a multimode fiber. *Opt. Express* **23**, 9109–9120 (2015).

13. Czarske, J. W., Haufe, D., Koukourakis, N. & Büttner, L. Transmission of independent signals through a multimode fiber using digital optical phase conjugation. *Opt. Express* **24**, 15128–15136 (2016).

14. Ryf, R. et al. Mode-multiplexed transmission over conventional graded-index multimode fibers. *Opt. Express* **23**, 235–246 (2015).

15. Franz, B. & Bulow, H. Experimental evaluation of principal mode groups as high-speed transmission channels in spatial multiplex systems. *IEEE Photon. Technol. Lett.* **24**, 1363–1365 (2012).

16. Zhu, L. et al. Orbital angular momentum mode groups multiplexing transmission over 2.6-km conventional multi-mode fiber. *Opt. Express* **25**, 25637–25645 (2017).

17. Mirhosseini, M. et al. Rapid generation of light beams carrying orbital angular momentum. *Opt. Express* **21**, 30196–30203 (2013).

18. Renninger, W. H. & Wise, F. W. Optical solitons in graded-index multimode fibres. *Nat. Commun.* **4**, 1719 (2013).

19. Perez-Garcia, B., López-Mariscal, C., Hernandez-Aranda, R. I. & Gutiérrez-Vega, J. C. On-demand tailored vector beams. *Appl. Opt.* **56**, 6967–6972 (2017).

20. Cuche, E., Marquet, P. & Depeursinge, C. Spatial filtering for zero-order and twin-image elimination in digital off-axis holography. *Appl. Opt.* **39**, 4070–4075 (2000).

21. Zhu, Z. et al. Single-shot direct tomography of the complete transverse amplitude, phase, and polarization structure of a light field. *Phys. Rev. Appl.* **12**, 034036 (2019).

22. Jang, M., Ruan, H., Zhou, H., Judkewitz, B. & Yang, C. Method for auto-alignment of digital optical phase conjugation systems based on digital propagation. *Opt. Express* **22**, 14054–14071 (2014).

23. Zhou, Y. et al. Sorting photons by radial quantum number. *Phys. Rev. Lett.* **119**, 263602 (2017).

24. Zhou, Y. et al. Hermite–Gaussian mode sorter. *Opt. Lett.* **43**, 5263–5266 (2018).

25. Ruffato, G. et al. A compact diffractive sorter for high-resolution demultiplexing of orbital angular momentum beams. *Sci. Rep.* **8**, 10248 (2018).

26. Thibos, L. N., Applegate, R. A., Schwiegerling, J. T. & Webb, R. Standards for reporting the optical aberrations of eyes. *J. Refract. Surg.* **18**, S652–S660 (2002).

27. Beijersbergen, M. W., Allen, L., Van der Veen, H. & Woerdman, J. Astigmatic laser mode converters and transfer of orbital angular momentum. *Opt. Commun.* **96**, 123–132 (1993).
